# The effects of tumor necrosis factor-alpha on systolic and diastolic function in rat ventricular myocytes

**DOI:** 10.1002/phy2.93

**Published:** 2013-09-17

**Authors:** David J Greensmith, Mahesh Nirmalan

**Affiliations:** 1 Unit of Cardiac Physiology, Institute of Cardiovascular Science, Manchester Academic Health Science Centre, Core Technology Facility46 Grafton Street, Manchester, M13 9NT, U.K.; 2 Critical Care Unit, Manchester Royal Infirmary and Manchester Medical School, University of ManchesterManchester, M13 9NT, U.K.

**Keywords:** Calcium, L-type calcium channel, myocyte, sarcoplasmic reticulum, tumor necrosis factor-alpha

## Abstract

The proinflammatory cytokine tumor necrosis factor-alpha (TNF-α) is associated with myocardial dysfunction observed in sepsis and septic shock. There are two fundamental components to this dysfunction. (1) systolic dysfunction; and (2) diastolic dysfunction. The aim of these experiments was to determine if any aspect of whole-heart dysfunction could be explained by alterations to global intracellular calcium ([Ca^2+^]_i_), contractility, and [Ca^2+^]_i_ handling, by TNF-α, at the level of the individual rat myocyte. We took an integrative approach to simultaneously measure [Ca^2+^]_i_, contractility and sarcolemmal Ca fluxes using the Ca indicator fluo-3, video edge detection, and the perforated patch technique, respectively. All experiments were performed at 37°C. The effects of 50 ng/mL TNF-α were immediate and sustained. The amplitude of systolic [Ca^2+^]_i_ was reduced by 31% and systolic shortening by 19%. Diastolic [Ca^2+^]_i_, myocyte length and relaxation rate were not affected, nor were the activity of the [Ca^2+^]_i_ removal mechanisms. The reduction in systolic [Ca^2+^]_i_ was associated with a 14% reduction in sarcoplasmic reticulum (SR) content and a 11% decrease in peak L-type Ca current (I_C__a-L_). Ca influx was decreased by 7% associated with a more rapid I_C__a-L_ inactivation. These data show that at the level of the myocyte, TNF-α reduces SR Ca which underlies a reduction in systolic [Ca^2+^]_i_ and thence shortening. Although these findings correlate well with aspects of systolic myocardial dysfunction seen in sepsis, in this model, acutely, TNF-α does not appear to provide a cellular mechanism for sepsis-related diastolic myocardial dysfunction.

## Introduction

Impaired cardiac function is a well-recognized feature of sepsis and septic shock (Kumar et al. 2001a; Court et al. 2002). Manifestations of this dysfunction fall into two broad categories. (1) Systolic dysfunction including impaired contractility and reduced ejection fraction and (2) diastolic dysfunction including ventricular dilation and impaired relaxation. It is well known that proinflammatory cytokines are implicated in the pathogenesis of sepsis, a notable example being tumor necrosis factor-alpha (TNF-α) (Kumar et al. 2001b). With regard to myocardial dysfunction, there is considerable evidence that TNF-α is implicated. Serum concentrations of TNF-α are elevated in sepsis (Waage et al. 1989; Casey et al. 1993), and animal models of sepsis (Michie et al. 1988), and a correlation between plasma concentrations of TNF-α and myocardial dysfunction has been established (Vincent et al. 1992; Herbertson et al. 1995; Kumar et al. 1996; Forfia et al. 1998). Furthermore, in vivo studies in dogs have demonstrated that injection of TNF-α can replicate the profile of systolic and diastolic dysfunction observed in sepsis (Natanson et al. 1989; Eichenholz et al. 1992; Walley et al. 1994; Murray and Freeman 1996). However, as most of these studies involved chronic exposure to TNF-α, it is difficult to elucidate, which, if any of the aspects of myocardial dysfunction are due to the direct action of TNF-α, or secondary to activation of other inflammatory cascades (Halliwell and Gutteridge 1999; Bayir 2005; Brandes and Kreuzer 2005) (e.g., generation of reactive oxygen species or nitric oxide species).

The purpose of this study was two-fold: (1) to determine if any of the above aspects of myocardial dysfunction caused by TNF-α could be explained by alterations to global [Ca^2+^]_i_ and contractility at the level of the single cardiac myocyte and (2) to explain any changes to global dysfunction by determining the effects of TNF-α on [Ca^2+^]_i_ handling. Because of the acute nature of TNF-α exposure, any effects observed would most likely be as a result of a direct action of TNF-α.

Others have studied the effects of TNF-α on the individual myocyte, however, the findings are contradictory and incomplete. Most have demonstrated a reduction in systolic [Ca^2+^]_i_ and shortening (Yokoyama et al. 1993; Goldhaber et al. 1996; Kumar et al. 1996; Amadou et al. 2002; Cailleret et al. 2004), although others have reported no effect on systolic [Ca^2+^]_i_ (Goldhaber et al. 1996) and even an increase (Amadou et al. 2002). The time frame of effect also varies considerably which has important implications not only in terms of relevance to pathology but also whether the reported effects are direct or indirect. Most report effects occurring progressively, over tens of minutes (Yokoyama et al. 1993; Goldhaber et al. 1996; Kumar et al. 1996; Amadou et al. 2002) though relatively acute effects (1 min) have been reported (Sugishita et al. 1999). Furthermore, some have demonstrated a biphasic effect of TNF-α, where an initial enhancement of systolic [Ca^2+^]_i_ precedes an ultimate decrease (Amadou et al. 2002; Cailleret et al. 2004). Despite these studies, the effects of TNF-α on the diastolic aspects of global [Ca^2+^]_i_ and contractility have been largely overlooked, as have the effects on the mechanisms of [Ca^2+^]_i_ handling.

The lack of consensus regarding the effects and time frame of effects of TNF-α on myocyte function may be as a result of the diverse range of models and techniques used. We present the first integrative approach in that the effects of TNF-α on global [Ca^2+^]_i_, contractility and [Ca^2+^]_i_ handling have been studied simultaneously. This has the advantage that correlations between findings are likely to be more robust.

These data suggest that TNF-α directly affects the L-type calcium channel (LCC) contributing to a reduced sarcoplasmic reticulum (SR) Ca content. This underlies a reduction in systolic [Ca^2+^]_i_ and thence shortening. However, we observed no effect on any aspect of diastolic function studied suggesting modifications to global [Ca^2+^]_i_, contractility and [Ca^2+^]_i_ handling at the level of the single myocyte by *direct* action of TNF-α can only be contributory factors to *systolic* whole-heart dysfunction.

## Methods

Unless stated, all chemicals used were obtained from Sigma Aldrich, Dorset, U.K.

### Cell isolation

Adult, male Wistar rats were humanely killed by stunning and cervical dislocation in accordance with the U.K. Animals (Scientific Procedures) Act 1986. Hearts were removed and the aorta cannulated for retrograde perfusion with a Ca-free solution containing (in mmol/L) NaCl: 134, HEPES: 10, Glucose: 11.1, NaH_2_PO_4_: 1.2, MgSO_4_: 1.2, KCl: 4, pH 7.34 with NaOH. Following a 10-min wash, Collagenase (Worthington Biochemical Cooperation, NJ) and type XIV protease (Sigma-Aldrich, Dorset, U.K.) were added at typical concentrations of 0.6 and 0.067 mg/mL, respectively, for a digest lasting around 7 min. For a 10-min wash, the solution was then switched to a low Ca solution containing (in mmol/L) NaCl: 115, HEPES: 10, Glucose: 11.1, NaH_2_PO_4_: 1.2, MgSO_4_: 1.2, KCl: 4, Taurine: 50, CaCl_2_: 0.1, pH 7.34 with NaOH. Cells were stored in this solution until use.

### Voltage clamp by perforated patch

Sarcolemmal currents were measured using the perforated patch technique under voltage clamp, using the switch clamp facility of the Axoclamp 2B voltage clamp amplifier (Axon instruments, CA). Microelectrodes with a typical resistance of 5 MΩ were filled with a caesium-based (to control outward currents) pipette solution containing (in mmol/L) CsCl: 20, Cs_3_CH_3_O_3_S: 125, NaCl: 10, HEPES: 10, MgCl_2_: 5, Cs_2_EGTA: 0.1, pH 7.2 with CsOH. Electrical access was achieved by addition of amphotericin B (240 μg/mL). *I*_Ca-L_ was activated using a 100-msec duration, 40 mV voltage step, from a holding potential of −40 mV applied at 0.5 Hz. An experimental solution containing (in mmol/L) NaCl_2_: 134, HEPES: 10, Glucose: 11.1, MgCl_2_: 1.2, CaCl_2_: 1, KCl: 4, probenecid; 2, pH 7.34 with NaOH was used for all experiments. Outward currents were inhibited by addition of BaCl_2_ (0.1 mmol/L) and 4-Aminopyridine (5 mmol/L). All experiments were carried out at 37°C.

### Measurement of [Ca^2+^]_i_ and cell shortening

[Ca^2+^]_i_ was measured by loading the cells with fluo-3 AM (5 μmol/L) for 10 min (Greensmith et al. 2010). Fluorescence was excited at 488 nm and emission measured at wavelengths greater than 525 nm (Greensmith et al. 2010). Fluorescence signals were calibrated and converted to reflect absolute Ca^2+^ concentrations using the following equation (Dibb et al. 2004):


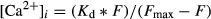


Where *K*_*d*_ is the dissociation constant of fluo-3 (864 nmol/L at 37°C), *F* is fluorescence, and *F*_max_ is maximal fluorescence obtained by damaging the cell at the end of each experiment. Background fluorescence was recorded and subtracted from the total fluorescence to derive *F*.

Diastolic myocyte length and the degree of systolic shortening were measured using a video edge detection system (Crescent Electronics Ltd, Sandy, Utah), calibrated against a graticule slide (Greensmith et al. 2010).

### Quantification of sarcolemmal Ca influx and sarcoplasmic reticulum Ca^2+^ content

Sarcolemmal Ca influx was quantified by integration of *I*_Ca-L_ activated by the voltage step described above. SR Ca^2+^ content was determined by integration of the inward sodium current evoked by rapid application of 10 mmol/L caffeine (Varro et al. 1993).

### Quantification of the activity of SERCA and NCX

To measure the combined activity of sarco-endoplasmic reticulum calcium ATPase (SERCA) and sodium-calcium exchanger (NCX), the rate of decay of the systolic [Ca^2+^]_i_ transient (k_sys_) was determined by fitting a single exponential. The activity of NCX alone was inferred from the rate of decay of the caffeine-evoked [Ca^2+^]_i_ transient (*k*_NCX_). The activity of SERCA alone (k_SERCA_) was calculated by subtraction of k_NCX_ from k_sys_ (Diaz et al. 2004; Dibb et al. 2004).

### Data analysis and statistics

For the experiments studying the chronic effects of TNF-α, we compared two groups; control (cells stored as above) and cells incubated with 25 ng/mL TNF-α for at least 1 h. For each parameter tested, mean data were determined by averaging 10 transients. Statistical significance was evaluated using a *t*-test (*P* < 0.05). For the experiments studying the acute effects of 50 ng/mL TNF-α, for each parameter tested, mean data were determined by averaging 10 transients from three experimental periods; control, initial TNF application (the first 20 sec of exposure), and prolonged TNF application (following ∼3 min of exposure). Repeated measures Analysis of variance (ANOVA), repeated measures ANOVA on ranks and paired *t*-tests were used to determine statistical significance (*P* < 0.05).

## Results

**Figure 1:**
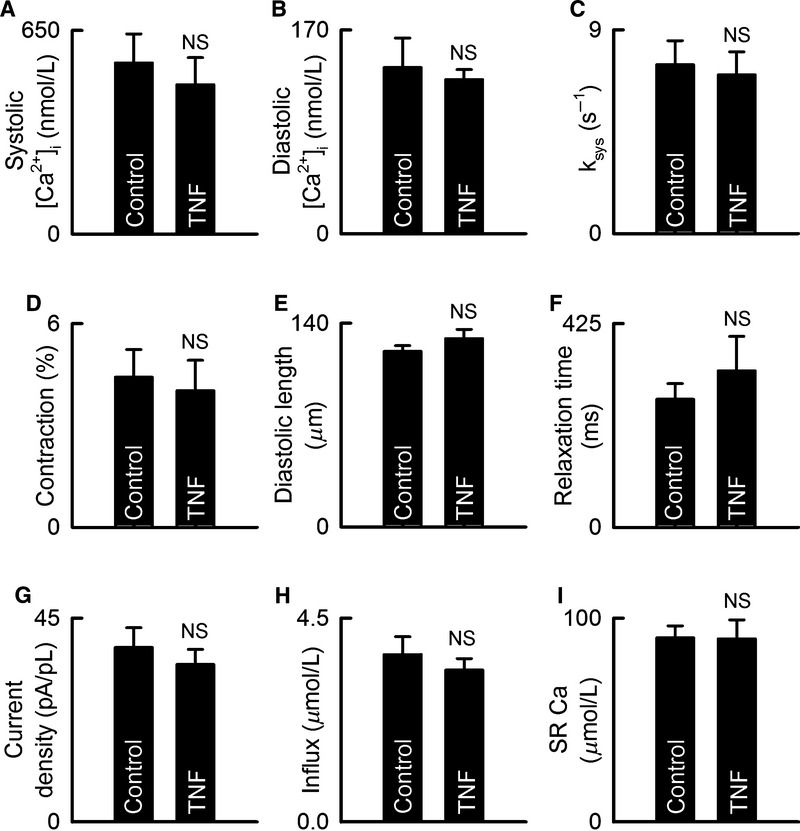
The chronic effects of 25 ng/mL tumor necrosis factor-alpha (TNF-α). In all panels, two groups are compared; control, and cells incubated with TNF-α for at least 1 h and show mean data for (A) [Ca^2+^]_i_ transient amplitude, (B) diastolic [Ca^2+^]_i_, (C) k_sys_, (D) systolic contraction, (E) diastolic cell length, (F) relaxation time (90–10% maximal), (G) peak I_C__a-L_ (normalized to cell volume), (H) Ca influx, (I) SR Ca content.

Our first experiments (Fig. 1) were designed to determine if relatively long-term exposure to TNF-α could produce any alterations to [Ca^2+^]_i,_ or contractility. Cells were incubated with 25 ng/mL TNF-α for at least 1 h. Incubation with TNF-α had no *significant* effect on the amplitude of systolic [Ca^2+^]_i_ (Control: 546 ± 92, TNF-α: 477 ± 86 nmol/L, *n* = 8 and 9, *P* = 0.59) or the degree of systolic shortening (Control: 4.4 ± 0.8, TNF-α: 4.0 ± 0.9%, *n* = 8 and 9, *P* = 0.75). Incubation with TNF-α also produced no effect on any parameter of diastolic function measured, including diastolic [Ca^2+^]_i_ (Control: 139 ± 24, TNF-α: 129 ± 8 nmol/L, *n* = 8 and 9, *P* = 0.47), the rate of systolic [Ca^2+^]_i_ removal (Control: 7.5 ± 1.1, TNF-α: 7.0 ± 1.0 sec^−1^, *n* = 8 and 9, *P* = 0.77), diastolic cell length (Control: 121 ± 4, TNF-α: 129 ± 6 μm, *n* = 8 and 9, *P* = 0.27) or the rate of relaxation (Control: 267 ± 33, TNF-α: 326 ± 72 msec, *n* = 8 and 9, *P* = 0.96). Furthermore, we observed no *significant* effect on peak *I*_Ca-L_ (Control: 39 ± 4, TNF-α: 35 ± 3 pA/pL, *n* = 8 and 9, *P* = 0.50), Ca influx (Control: 3.70 ± 0.39, TNF-α: 3.35 ± 0.25 μmol/L, *n* = 8 and 9, *P* = 0.46), or SR Ca content (Control: 90 ± 6, TNF-α: 90 ± 9 μmol/L, *n* = 7 and 9, *P* = 0.97).

**Figure 2:**
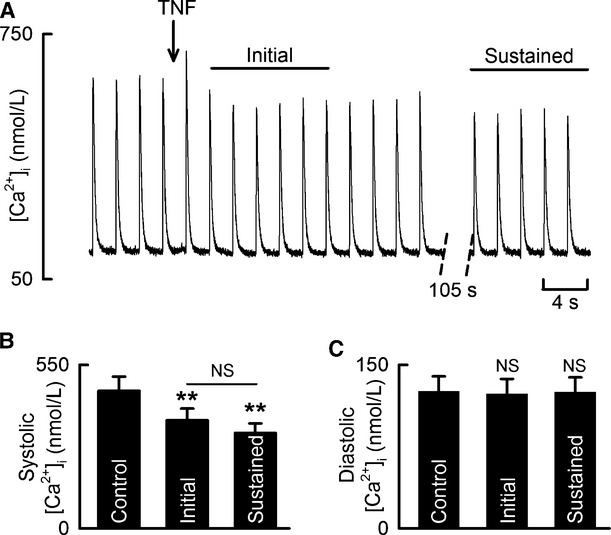
The effects of 50 ng/mL TNF-α on [Ca^2+^]_i_. (A) specimen record showing [Ca^+^]_i_ transients. (B and C), respectively; mean [Ca^2+^]_i_ transient amplitude and diastolic [Ca^2+^]_i_. In all panels, the periods compared are control, initial TNF-α application (within 20 sec), and sustained TNF-α application (∼3 min).

**Figure 3:**
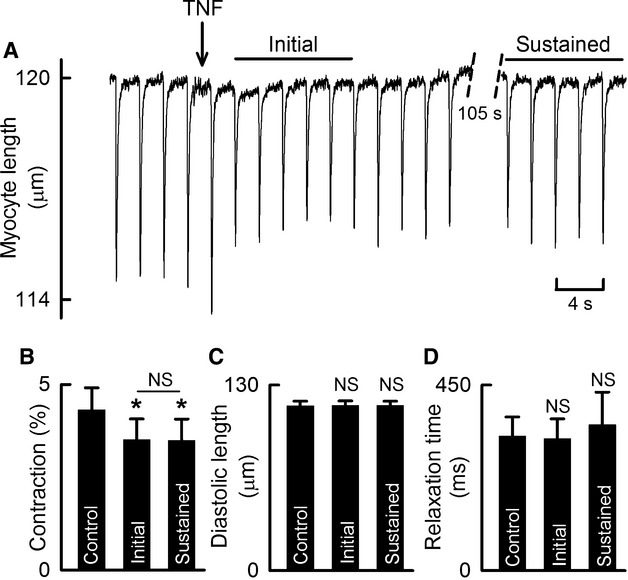
The effects of 50 ng/mL TNF-α on contractility. (A) Specimen record showing contractility transients. (B) Mean systolic shortening. (C) Mean diastolic cell length. (D) Mean relaxation time (90–10% of maximal systolic shortening). In all panels, the periods compared are control, initial TNF-α application (within 20 sec), and sustained TNF-α application (∼3 min).

Next, we studied the effects of acute exposure to 50 ng/mL TNF-α in order to (1) see if a larger concentration could produce an effect, and to (2) determine the *direct* effects of TNF-α. The effects of 50 ng/mL TNF-α on systolic [Ca^2+^]_i_ and myocyte contraction were immediate and sustained (Figs. 2, 3). In most myocytes, the application of TNF-α was associated with an immediate augmentation of the [Ca^2+^]_i_ transient amplitude, associated with an increase in systolic shortening. However, this lasted only one beat and was followed by a rapid reduction in systolic [Ca^2+^]_i_ occurring within seconds of exposure. Following a typical exposure of 3 min, systolic [Ca^2+^]_i_ was reduced by 31% (Control: 462 ± 48, TNF-α: 321 ± 32 nmol/L, *n* = 8, *P* < 0.005). This was associated with a 19% reduction in systolic shortening (Control: 4.3 ± 0.6, TNF-α: 3.5 ± 0.6%, *n* = 9, *P* < 0.05). Diastolic [Ca^2+^]_i_ (Control: 126 ± 14, TNF-α: 125 ± 13 nmol/L, *n* = 8, *P* = 0.7), myocyte length (Control: 116 ± 3, TNF-α: 116 ± 3 μm, *n* = 9, *P* = 0.6) and relaxation time (90–10% of maximal shortening: Control: 326 ± 46, TNF-α: 354 ± 79 msec, *n* = 9, *P* = 0.6) were unaffected by exposure to TNF-α. After TNF-α exposure, we attempted a wash period to study the reversibility of the effects. However application of caffeine to quantify SR Ca following TNF-α usually resulted in cell death. Consequently, the reversibility of the above effects could not be studied. In time-dependent controls (data not shown), no effects were observed over equivalent time periods to those of TNF-α exposure.

**Figure 4:**
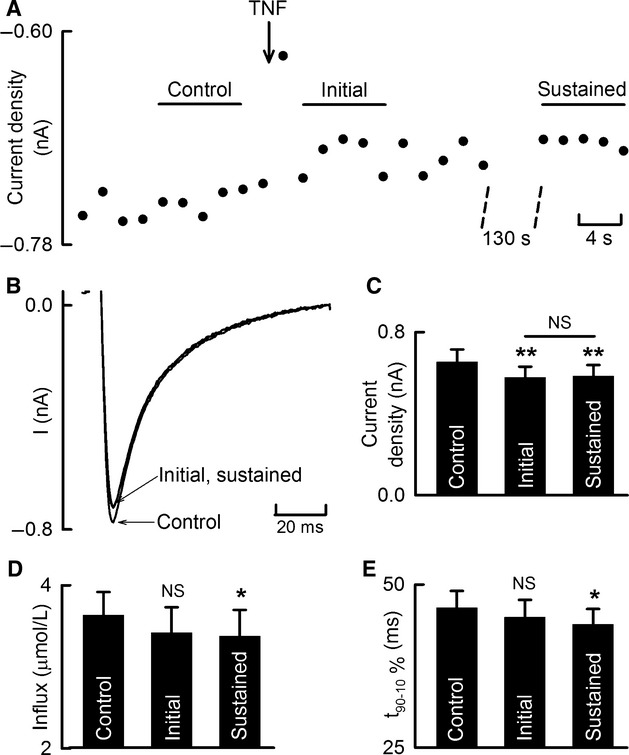
The effects of 50 ng/mL TNF-α on the LCC and I_C__a-L_. (A) Specimen record showing the time course of effect on peak I_C__a-L_. (B) Specimen current traces from the periods highlighted in A. (C) Mean peak I_C__a-L_. (D) Mean Ca influx on the I_C__a-L_. (E) Mean LCC inactivation time (90–10% of peak I_C__a-L_). In all panels, the periods compared are control, initial TNF-α application (within 20 sec), and sustained TNF-α application (∼3 min).

We next investigated whether changes to [Ca^2+^]_i_ handling played a role in the decrease in systolic [Ca^2+^]_i_, starting with the LCC. These data were recorded simultaneously with those above. Figure 4 summarizes the effects of TNF-α on *I*_Ca-L_. Peak *I*_Ca-L_ was reduced by 11% (Control: 0.65 ± 0.06, TNF-α: 0.58 ± 0.05 nA, *n* = 9, *P* < 0.05). Ca influx via the LCC was reduced by 7% (Control: 3.64 ± 0.28, TNF-α: 3.38 ± 0.32 μmol/L, *n* = 9, *P* < 0.05). LCC inactivation time (as determined by the time taken for *I*_Ca-L_ to decay from 90% to 10% of maximal) was reduced by ∼6% (Control: 46.45 ± 2.55, TNF-α: 43.89 ± 2.35 msec, *n* = 9, *P* < 0.05) suggesting a direct inhibition of the LCC.

**Figure 5:**
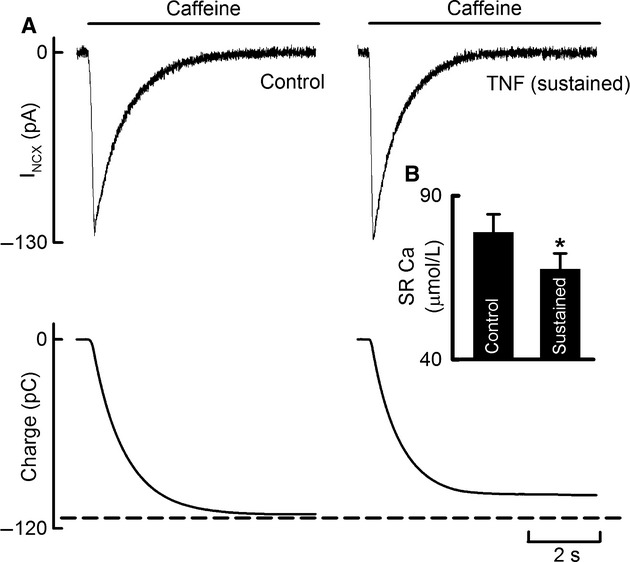
The effects of 50 ng/mL TNF-α on SR Ca. (A) Specimen records showing caffeine-evoked inward sodium currents (generated by the NCX-mediated removal of cytoplasmic Ca) (top) and the integral of those currents (below). B (inset); mean SR Ca content. The periods compared are control and sustained TNF-α application (∼3 min).

To determine if a decrease in SR Ca played a role in the decrease in systolic [Ca^2+^]_i_, SR Ca content was quantified by integration of caffeine-evoked inward sodium current (Fig. 5). After ∼3 min exposure to TNF-α, mean SR Ca^2+^ content was reduced by 14% (Control: 79 ± 5; TNF-α: 68 ± 5 μmol/L, *n* = 7, *P* < 0.05).

**Figure 6:**
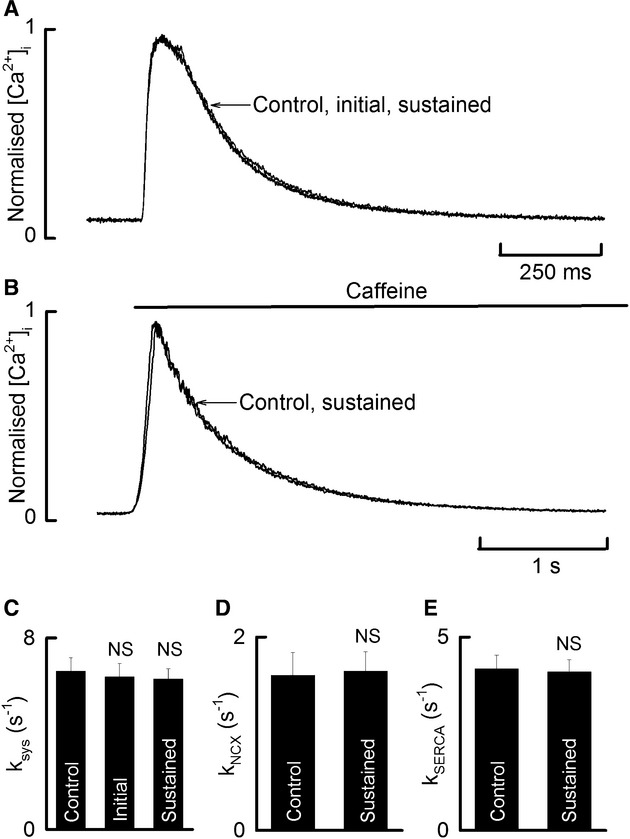
The effects of 50 ng/mL TNF-α on the [Ca^2+^]_i_ removal mechanisms. (A) Specimen records showing normalized systolic [Ca^2+^]_i_ transients. (B) Specimen records showing normalized caffeine-evoked [Ca^2+^]_i_ transients. (C) Mean k_sys_. (D) Mean k_NCX_. (E) mean k_SERCA_.

To further investigate diastolic function in the presence of TNF-α, the activity of the [Ca^2+^]_i_ removal mechanisms were quantified (Fig. 6). TNF-α had no effect on the activity of the combined [Ca^2+^]_i_ removal mechanisms (*k*_sys_ – Control; 6.62 ± 0.56, TNF-α; 6.29 ± 0.42 sec^−1^, *n* = 8, *P* = 0.1), NCX (*k*_NCX_ – Control; 1.60 ± 0.23, TNF-α; 1.65 ± 0.20 sec^−1^, *n* = 5, *P* = 0.4) or SERCA (*k*_SERCA_ – Control; 4.18 ± 0.35, TNF-α; 4.10 ± 0.32 sec^−1^, *n* = 5, *P* = 0.5).

## Discussion

### The effects of chronic TNF-α exposure

In our hands, incubation with 25 ng/mL TNF-α produced no *significant* effect on any measured parameter of global systolic or diastolic function (Fig. 1). Nor did we observe any effect on the major [Ca^2+^]_i_ handling mechanisms. Subsequently, we studied the effects of acute exposure to TNF-α. Our aim was to (1) determine if a larger concentration of TNF-α could produce an effect, and (2) determine the *direct* effects of TNF-α. With regard to (1), based on the lack of effect described above, we increased the TNF-α concentration to 50 ng/mL.

The plasma concentration of TNF-α reported in patients with sepsis is around 1 ng/mL. However, the action of cytokines such as TNF-α can be paracrine in nature so plasma concentrations underestimate the relevant concentrations at the site of action. Also, the use of 50 ng/mL TNF-α is typical of other experimenters in the field (Kumar et al. 2001a; Court et al. 2002).

### The acute effects of TNF-α on systolic [Ca^2+^]_i_ and contractility

Our data demonstrating the effects of 50 ng/mL TNF-α confirm previously reported findings that a reduction in *cellular* systolic [Ca^2+^]_i_ may, at least partially, contribute to TNF-α-induced reduction in myocardial contractility (Eichenholz et al. 1992; Walley et al. 1994). In most cells (Figs. 2, 3), we observed an initial augmentation of systolic [Ca^2+^]_i_ and contraction. Although technically this agrees with the biphasic effect of TNF-α seen by others (Cailleret et al. 2004), in our hands this effect lasted only one beat before rapidly developing into a *reduced* systolic [Ca^2+^]_i_ and % shortening. Although agreeing qualitatively with the ultimate effect seen in most previous studies (Yokoyama et al. 1993; Goldhaber et al. 1996; Sugishita et al. 1999; Amadou et al. 2002; Cailleret et al. 2004), the onset of the reduction in systolic [Ca^2+^]_i_ observed by us is *the* most rapid reported, possibly as a result at working at physiologically relevant temperatures.

### The acute effects of TNF-α on diastolic [Ca^2+^]_i_ and relaxation

Although some studies have shown that TNF-α has no effect on global diastolic function (Yokoyama et al. 1993; Goldhaber et al. 1996), the effects on other diastolic functions such as the [Ca^2+^]_i_ removal mechanisms, have been largely overlooked. This study has added to the current body of knowledge by demonstrating that, in our hands acute exposure to 50 ng/mL TNF-α has no effect on both myocyte relaxation and the activity of the [Ca^2+^]_i_ removal mechanisms (Figs. 3, 6). The absence of direct effects on myocyte relaxation or the [Ca^2+^]_i_ removal mechanisms is in fact surprising given the fact that TNF-α has been shown to inhibit phosphorylation of phospholamban (Yokoyama et al. 1999). Also, the fact we observe no changes to resting myocyte length at constant diastolic [Ca^2+^]_i_ suggests a lack of effect on myofilament sensitivity, a phenomenon which *has* been reported by others (Goldhaber et al. 1996). Furthermore, this finding suggests that the reduction in systolic shortening occurs directly as a result of reduced systolic [Ca^2+^]_i_ rather than as a result of or in combination with a direct effect on the myofilaments.

### The acute effects of TNF-α on the L-type calcium channel

There is currently little consensus as to the effects of TNF-α on the LCC and thus *I*_Ca-L_ (Yokoyama et al. 1993; Krown et al. 1995; Sugishita et al. 1999). The integrative approach used in this study is advantageous as it has allowed us to study the effects of TNF-α on the *I*_Ca-L_ simultaneously with global myocyte function. We suggest therefore that correlations between observed effects are more robust. We observed a small, but significant reduction in peak *I*_Ca-L_ (Fig. 4) which itself may contribute to the reduction in systolic [Ca^2+^]_i_ (Eisner et al. 2000). Under physiological conditions, one may expect a reduction in systolic [Ca^2+^]_i_ to be associated with delayed LCC inactivation and thence enhanced Ca influx (Trafford et al. 1997). Following TNF-α exposure we observed a small but significant increase in the rate of LCC inactivation as indicated by the reduction in the time taken for *I*_Ca-L_ to decay from 90% to 10% of peak. This actually results in a 7% decrease in Ca influx. This suggests a direct action of TNF-α on the LCC.

### What underlies the reduction in systolic [Ca^2+^]_i_?

Others have suggested that the effects of TNF-α on the LCC are directly responsible for the reduction in systolic [Ca^2+^]_i_ (Krown et al. 1995). However, in our hands, given the relatively small effect of TNF-α on peak *I*_Ca-L_¸ it is unlikely that this alone can account for the reduction in systolic [Ca^2+^]_i_ (Bassani et al. 1995). To seek an alternative explanation, we quantified the effect of TNF-α on SR Ca content (Fig. 5). The amplitude of systolic [Ca^2+^]_i_ is proportional to the third power of SR Ca content (Trafford et al. 2000). Following TNF-α exposure, systolic [Ca^2+^]_i_ was seen to fall to 69% of control values. The cube root of this decrease predicts that SR Ca content would need to fall by 12% to account for the observed decrease in systolic [Ca^2+^]_i_. We observe a 14% decrease in SR Ca which suggests that this alone can account for the reduction in systolic [Ca^2+^]_i_.

### What mechanisms reduce sarcoplasmic reticulum Ca content?

TNF-α had no effect on the activity of SERCA (Fig. 6), which can, to some extent, modulate SR Ca (Bode et al. 2011). Can the reduced Ca influx on *I*_Ca-L_ be responsible for the decreased SR Ca content? First, we must consider that the *I*_Ca-L_ plays two roles in Ca handling: (1) it triggers SR Ca release in a graded fashion such that the greater the peak *I*_Ca-L_ the greater fractional release and thence systolic [Ca^2+^]_i_ (Bassani et al. 1995), and (2) it loads the cytoplasm with Ca, increasing Ca availability to SERCA facilitating SR loading (Fabiato 1985). Trafford et al. (2001) have shown that increasing *I*_Ca-L_ (both in terms of peak current and influx) increases systolic [Ca^2+^]_i_ but has no effect on SR Ca (Trafford et al. 2001). This is because although an increase in Ca influx facilitates Ca loading thus *increasing* SR Ca content, the potentiation of systolic [Ca^2+^]_i_ by the increased trigger leads to enhanced efflux *decreasing* SR content (Trafford et al. 2000; Eisner et al. 2013). The converse is true if *I*_Ca-L_ is decreased. The effect of the roles explained above effectively balance out and SR Ca remains constant.

However, in our hands, although TNF-α reduces both peak *I*_Ca-L_ and influx, the effect on peak *I*_Ca-L_ is small. Under these conditions, the effects of reduced loading may be greater than the effect of the reduction in *trigger-dependent* systolic [Ca^2+^]_i_ leading to a decrease in SR Ca. Can the 7% decrease in Ca influx therefore account for the 14% reduction in SR Ca? If we assume the decrease in systolic [Ca^2+^]_i_ is entirely due to a decrease in SR Ca caused by reduced Ca influx, and, we assume the reduction in systolic [Ca^2+^]_i_ is proportional to the decrease in Ca influx and thence efflux, a 31% decrease in Ca influx would be required. Therefore, although the reduction in Ca influx by TNF-α may contribute to the reduced SR Ca^2+^ content, it cannot *fully* explain it.

What other mechanism could contribute to SR Ca loss? Duncan et al. (2010) reported that TNF-α increases the open probability of the ryanodine receptor (RyR); which is known to contribute to SR Ca loss (Zima et al. 2010). The first beat augmentation of systolic [Ca^2+^]_i_ seen in this study is consistent with an increased RyR open probability (Trafford et al. 2000).

### Summary and clinical relevance

We have shown that, by a reduction in SR Ca, TNF-α reduces systolic [Ca^2+^]_i_ and shortening at the level of the individual myocyte. Although inhibition of the LCC may *contribute* directly to this, we suggest it is the subsequent depletion of SR Ca that is the primary mechanism. In this study, TNF-α had no effect on any measured parameter of diastolic function. We suggest therefore that the *direct* effects of TNF-α on cell [Ca^2+^]_i_ and contractility could contribute to whole-heart systolic dysfunction observed in sepsis. However, these data suggest that the *direct* effects of TNF-α on cell [Ca^2+^]_i_ and contractility do not contribute whole-heart diastolic dysfunction.
